# Single motor unit firing behavior in the right trapezius muscle during rapid movement of right or left index finger

**DOI:** 10.3389/fnhum.2014.00881

**Published:** 2014-11-03

**Authors:** Karen Søgaard, Henrik B. Olsen, Anne K. Blangsted, Gisela Sjøgaard

**Affiliations:** ^1^Department of Sports Science and Clinical Biomechanics, University of Southern Denmark, OdenseDenmark; ^2^Outcome Europe, St-PrexSwitzerland

**Keywords:** computer mouse double clicking, single motor units, trapezius, doublets, contralateral activity

## Abstract

**Background:** Computer work is associated with low level sustained activity in the trapezius muscle that may cause development of trapezius myalgia. Such a low level activity may be attention related or alternatively, be part of a general multi joint motor program providing stabilization of the shoulder joint as a biomechanical prerequisite for precise finger manipulation. This study examines single motor unit (MU) firing pattern in the right trapezius muscle during fast movements of ipsilateral or contralateral index finger. A modulation of the MU firing rate would support the existence of a general multi joint motor program, while a generally increased and continuous firing rate would support the attention related muscle activation.

**Method:** Twelve healthy female subjects were seated at a computer work place with elbows and forearms supported. Ten double clicks (DC) were performed with right and left index finger on a computer mouse instrumented with a trigger. Surface electromyographic signals (EMG) was recorded from right and left trapezius muscle. Intramuscular EMG was recorded with a quadripolar wire electrode inserted into the right trapezius. Surface EMG was analyzed as RMS and presented as %MVE. The intramuscular EMG signals were decomposed into individual MU action potential trains using a computer algorithm based on signal shape recognition and manual editing. Instantaneous firing rate (IFR) was calculated as the inverse of each inter-spike interval (ISI). All ISI shorter than 20 ms were defined as doublets. For all MU IFR was spike triggered averaged across the 10 DC to show the modulation during DC as well as for calculation of the cross correlation coefficient (CCC).

**Results:** All subjects showed surface EMG activity in both right and left trapezius ranging from 1.8 %MVE to 2.5 %MVE. Regarding intramuscular EMG during right hand DC a total of 32 MUs were identified. Four subjects showed no MU activity. Four showed MU activity with low mean firing rate (MFR) with weak or no variations related to the timing of DC. Four subjects showed firing patterns with large modulation in IFR with a clear temporal relation to the DC. During left hand DC 15 MUs were identified in four subjects, for two of the subjects with IFR modulations clearly related to DC. During both ipsi- and contralateral DC, doublets occurred sporadically as well as related to DC

**Conclusion:** In conclusion, DC with ipsi- and contralateral fast movements of the index finger was found to evoke biomechanically as well as attention related activity pattern in the trapezius muscle. Doublets were for three of the subjects found as an integrated part of MU activation in the trapezius muscle and for one subject temporarily related to DC.

## INTRODUCTION

During data entry on the computer low static activity in the upper trapezius muscle has been reported even at an optimal ergonomic work station with full elbow support and no obvious biomechanical need (Kitahara et al., 2000; Thorn et al., 2007). For the uppermost clavicular subpart of the trapezius muscle the activity may be due to the need for postural neck extension and/or gaze stabilization during computer work. In contrast, the acromial subpart that is most prone for myalgia is mostly involved in scapular stabilization and accordingly, presents with a low activity level in the resting upright sitting position. However, in spite of the same modest biomechanical demand during computer work in the same position, it often shows activity above resting level (Sjøgaard et al., 2006). This low level but sustained muscle activity has been associated with development of trapezius myalgia and it has been speculated, whether it is mainly related to the mental demand of attention. (Sjøgaard et al., 2000; Wærsted, 2000; Zennaro et al., 2003). An alternative suggestion is that the trapezius muscle plays a role in a general multi joint motor program providing stabilization of the shoulder joint, as this in many situations may be a biomechanical prerequisite for precise finger manipulations (Alexander and Harrison, 2003). During computer work the double click (DC) task on the computer mouse is an example of such a specialized finger manipulation demanding a small but fast flexion and extension in the metacarpophalangeal joint of the index finger. Earlier studies have shown that during this dynamic task, modulated firing patterns often involving doublets are evoked in the extensor digitorum communis muscle (Sjøgaard et al., 2001; Søgaard et al., 2001). If the computer work related trapezius activation is mainly caused by attention demand, a regular continuous firing pattern in both ipsi- and contralateral shoulder muscles would be expected during the dynamic DC task. However, if activation is related to biomechanical demands, the firing pattern in the stabilizing trapezius muscle would show task related modulation during the actual finger task.

The aim of the study was to examine single motor unit (MU) firing pattern in the right trapezius muscle during fast movements of the ipsilateral or contralateral index finger. A mechanical task related modulation of the MU firing would support the existence of a general multi joint motor program, while a bilateral generally increased continuous firing rate would support the attention related motor control.

## MATERIALS AND METHODS

### SUBJECTS AND PROCEDURE

Twelve healthy female subjects volunteered in the study after giving informed consent [Age: 24 (19–38) years, height 1.70 (1.62–1.78) m, and 63 (56–74) kg]. The participants reported no discomfort in the upper body regions within the week prior to testing. Eleven subjects reported to be right handed, while one subject (ID 11) reported to be left handed. All subjects were experienced computer users in their daily work and operated the computer mouse with their right hand. Therefore, in the present study the MU firing pattern was investigated for the right trapezius muscle for all subjects. During the experiment the subjects were seated upright in a height adjustable chair with forearms and elbows fully supported on the surface of a height adjusted table.

The subject performed ten DC interspersed by approximately two s on a computer mouse instrumented with a custom built trigger under the left mouse key. The task was intended to simulate the normal task of double clicking during computer work in order to evoke a function seen on the computer screen and thus was not particularly attention demanding. The subject was instructed to pay attention and follow the visual cue of a color change on the screen throughout the 10 DC sequence, but no particular emphasis on timing or speed was expressed. In a balanced order the subjects performed the DC task with the left as well as the right index finger. During the recordings no auditory or visual feedback of the MU activity was given to the subject. The study was approved by the local ethical committee (KF 01–298/00).

### MEASUREMENTS

#### Surface EMG

**FIGURE 1:**
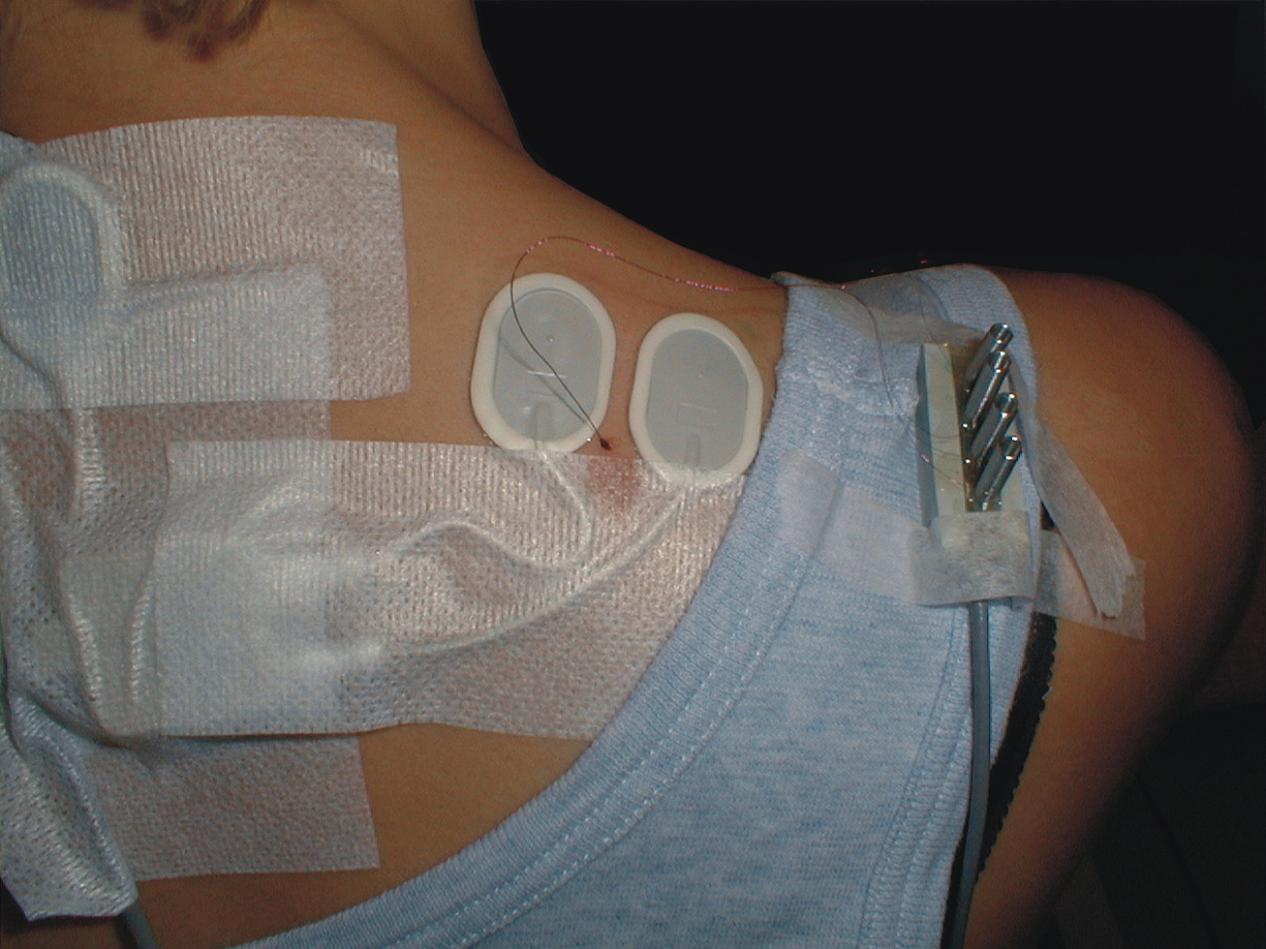
**Surface electrode configuration and also including the wire electrode for the right trapezius muscle.** Note that the intramuscular quadripolar wire electrode is inserted at a 30∘ angle and at a distance so the location of the tip of the wire is estimated to be between the surface electrodes and in the recorded muscle volume.

Electromyographic signals (EMG) were recorded bilaterally from the upper trapezius muscles using bipolar surface electrodes (Ag–AgCl electrodes, type 72001-K, Medicotest, Denmark). The center of each pair of electrodes was placed 2 cm medial to the midpoint between the seventh cervical vertebrae and the lateral end of acromion. The inter-electrode distance was 20 mm. In **Figure 1** the electrode configuration also including the wire electrode is shown for the right trapezius muscle. The EMG signal was amplified, low-pass filtered (eighth order Butterworth filter, cut-off 400 Hz), and sampled on a computer with a sampling frequency of 1024 Hz. The signals were visually checked and high-pass filtered (cut-off 10 Hz), full-wave rectified, and root mean-square converted within windows of 100 ms duration. The resting EMG signal was recorded during 5 s of instructed rest in the same postural position as during the DC recordings. Visual feedback was provided on the screen to help the subject eliminate visible EMG activity, and the resting EMG amplitude was quadratically subtracted from all other EMG signals. For normalization, the maximal EMG amplitude obtained during three maximal voluntary isometric contractions was used. The contraction was performed bilaterally in the position of 90∘ shoulder flexion with resistance just proximal to the elbow. The maximal EMG amplitude (MVE) was calculated as the highest mean EMG amplitude obtained with a 1-s window moving in steps of 100 ms.

### INTRAMUSCULAR EMG

**FIGURE 2:**
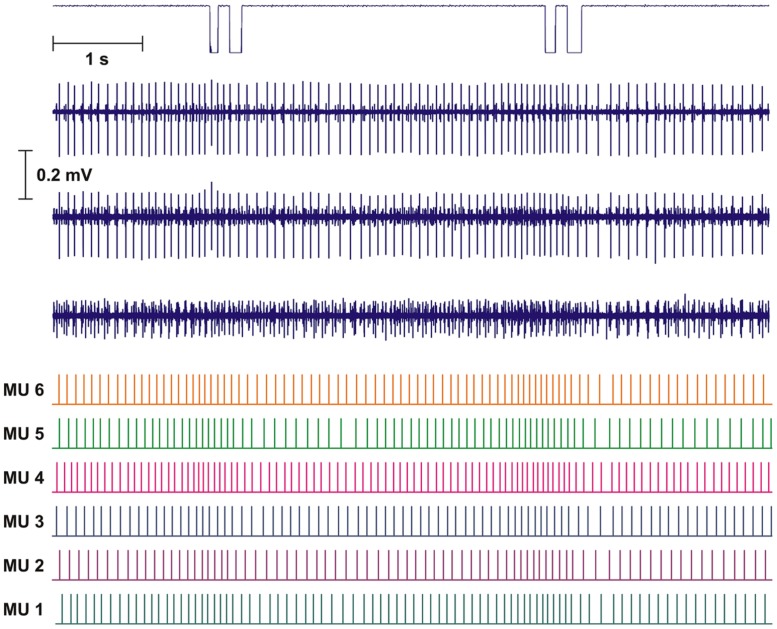
**Recording and analysis of two double DC.** Upper part shows mouse key trigger signal and the three intramuscular bipolar recordings of the raw electromyographic signals (EMG). The lower part shows the result of the decomposition presented as the inter firing bar plot for each of the identified motor unit (MUs). Based on the distance of the bars the instantaneous firing rate (IFR) for each MU was calculated.

Intramuscular EMG was recorded from the right trapezius muscle. A quadripolar wire electrode (Silvergold, Type 1, EGG Company, Tokyo, Japan) with a hooked end was inserted 3–4 cm into the muscle at an angle of 30∘ to the skin surface with a 27 gage cannula, that was subsequently withdrawn leaving the recording site of the wire in the trapezius approximately 2 cm medial to the midpoint between acromion and C7, i.e., in close spatial vicinity of the surface electrode recording site (See **Figure 1**). The wire consisted of four 50 μm urethane coated silvergold wires embedded in an epoxy coating. The distance in the exposed wire endings were 150–200 μm in a rectangular configuration. The four wires were combined to provide three highly selective bipolar recordings of micro MU action potentials (see **Figure 2**, upper part). All signals were analog filtered with a 10–10 kHz bandpass filter and A/D converted and stored on a computer with a sampling rate of 50 kHz for off-line analysis.

Before and after each DC recording it was tested, that the position of the wire electrode was in the vicinity of low threshold trapezius MUs by asking the subject to slightly lift the forearms from the table. The scheduled timing of each DC was given by a color change on the screen in front of the subject, and the actual time was recorded by a custom build computer mouse instrumented with a trigger under the left mouse button.

### DATA ANALYSIS

The intramuscular EMG signals were decomposed into individual MU action potential trains (MUAPT) using a computer algorithm based on shape recognition from the three bipolar channels and manual editing. The program is able to handle superimpositions of MUs by composing a comparison signal based on the waveform shape of available active MUs potentials and time shifting these. When a match in shape with the real superimposed signal is available, the combination and timing of MUs in the composed signal is suggested to the operator. (For details see Olsen and Søgaard, 1999; Olsen et al., 2001).

The performance of the program, when operated by the same highly skilled operator as in the present study, was evaluated by decomposition of 18 synthetic signals presenting different degrees of difficulties. The program showed a high accuracy of classification (always above 96%, in 17 out of 18 cases above 99%) and robustness to handling of irregular firing statistics, superimposed action potentials and shape changing of the same MU as well as MU shape similarity between different MU (Farina et al., 2001).

The program is highly interactive and provides excellent visual guidance for manual classification as well as automatically suggested best choice guided by high cross correlation and lowest normalized error power.

The results of the automatic decomposition algorithm were only accepted after detailed manual checking and editing, with calculated parameters as useful guidelines. Results are presented as inter firing interval (IFI) bar plots giving median firing rate for the time period presented (see **Figure 2**, lower part).

Instantaneous firing rate was calculated as the inverse of each IFI. Continuous activity was defined as MUAPT with no IFI longer than three times the median IFI of the train. Mean firing rate (MFR) was calculated as mean of IFR and presented as pulses per second and SD. IFIs shorter than 20 ms were defined as doublets, these were registered but the inter spike interval in the doublet was disregarded in the calculation of MFR, thus counting the doublet as one firing.

A more robust description of the shape of IFR modulation during a DC for each MUAPT was evaluated by spike triggered averaging the IFR across the 10 DCs using the trigger signal for alignment of 1 s pre and 1 s post the time of first click in each DC registered by the computer mouse button. For the spike triggered average all MU with continuous firing rate within the 2 s time interval around DC were included.

Finally, as a measure of the common drive and using the spike triggered averaging of the IFR over the 10 DC, the normalized cross correlation coefficients (CCC) between the averaged IFR profiles for MUs in each recording were calculated (De Luca et al., 1982, 2009). Before cross correlation of the averaged IFR for MU pairs the DC component of the MFR were removed using a digital zero-phase band pass filter from 0.75 to 12 Hz (De Luca et al., 2009). Signals were time shifted from one s back in time to one s forward in time to find the peak CCC and the time lag for the peak CCC.

### STATISTICS

Differences in surface EMG were tested with a two way repeated measures ANOVA for interaction between left/right trapezius and contralateral/ipsilateral DC. The MU firing pattern associations and temporal relations to DC were determined as “no,” “weak,” and “strong” subjectively, based on visual examination of individual IFR profiles, rather than assessed quantitatively. CCC among averaged IFR profiles for MUs from recording sites with modulating versus constant firing rates were too few for a proper statistical comparison and are presented only as graphs and descriptive data.

## RESULTS

### SURFACE EMG

During the 10 DCs surface EMG revealed activity above resting level for all 12 subjects in both right and left shoulder. During right hand DC the activity in right and left trapezius was a mean of 2.4 (1.6) %MVE and 1.8 (1.0) %MVE, respectively. During left hand DC the activity in right and left trapezius was a mean 2.0 (1.1) %MVE and 2.5 (1.1) %MVE, respectively. The two way ANOVA showed no significant interaction between ipsi/contralateral DC and left/right trapezius (*P* = 0,134) or any main effect for ipsi/contralateral DC (*P* = 0,989) or left/right Trap (*P* = 0,726).

### INTRAMUSCULAR EMG

An overview of the results for all subjects showing single MU recordings during right and left hand DC is shown in **Table 1**. The brief low level contraction of the trapezius just before and after the recording showed, that the tip of the wire was in a position, where potentially active MUs were in the vicinity of the tip of the intramuscular wire for all subjects. During the right and left hand DC task eight and four subjects, respectively, showed single MU activity. The three subjects (ID 5, 7, and 11) who did not show single MU activity during either right or left hand DC, showed a similar %MVE recorded from surface EMG as the subjects showing single MU activity.

**Table 1:** Overview of identified MU.

Subject ID	MU (N)	Continuously active MU throughout recording (N)	Range of MFR (pps)	Range of SD in MFR	Range of number of firings (N)
**Ipsilateral double click (DC)**
1	5	4	10.4-11.7	1.5-1.8	256-285
3	3	1	11.1	1.7	432
4*	8	2	9.6-12.2	2.2-2.8	278-346
6	2	2	8.6-9.8	0.9-1.1	169-170
8*	5	4	11.6-13.1	2.4-3.2	210-236
9	3	2	7.4-8.1	1.0-1.2	191-207
10	2	2	8.0-8.6	1.0-1.5	344-381
12*	4	1	13.4	2.3	529
**Contralateral DC**
2*	1	0	-	-	-
4	2	0	-	-	-
8	5	5	13.3-16.1	1.9-3.0	311-376
12*	7	5	10.1-13.0	1.6-2.3	468-603

*The asterisk indicates recordings including double discharges.*

While only 18 and 10 MU showed continuous firing for the whole recording (**Table 1**), 28 and 14 MUs fulfilled the criteria of continuous firing for the 2 s period surrounding the ipsilateral and contralateral DCs, respectively, and were used for spike triggered averaging IFR and for calculating CCC (**Table 2**). Peak CCC was a mean of 0.76 with a range between subjects from 0.40 to 0.93; while the mean time lag was zero with a range from -39 to 30 ms (See **Table 2**).

**Table 2:** Overview of CCC during DC for continuously active MU.

	Continuously active ±1 s around DC (N)	Mean peak CCC	Time lag (Range) (s)
**Ipsilateral DC**
ID 1	4	0.70 (0.64-0.77)	0.004 (-0.009-0.010)
ID 3	3	0.77 (0.73-0.81)	0.006 (0.005-0.007)
ID 4	7	0.78 (0.65-0.89)	0.007 (-0.023-0.042)
ID 6	2	0.73	-0.039
ID 8	5	0.90 (0.86-0.96)	0.001 (-0.006-0.005)
ID 9	2	0.40	0.030
ID 10	2	0.78	0.010
ID 12	3	0.91 (0.84-0.96)	0.008 (-0.005-0.016)
**Contralateral DC**
ID 2	1	-	-
ID 4	1	-	-
ID 8	5	0.68 (0.71-0.76)	0.001 (-0.004-0.007)
ID 12	7	0.93 (0.89-0.97)	0.008 (-0.018-0.032)

### IPSILATERAL DC

During the right index finger DC, eight subjects showed MU activity in the right trapezius muscle, and all had one or more continuously active MUs. As a total 32 MUs were identified and 18 MUs showed continuous activity with MFR ranging from 7.4 to 13.4 pps. The number of identified firings in the MUAPT ranged from 169 to 529 discharges.

**FIGURE 3:**
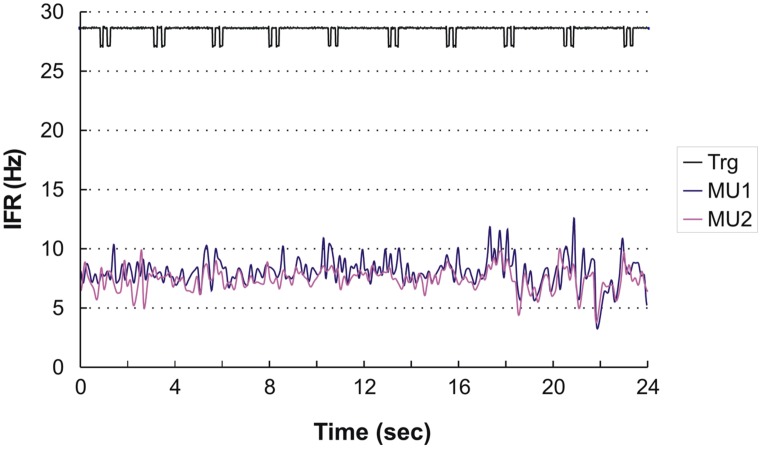
**Instantaneous firing rate plot showing the firing pattern of the identified MU in subject 9 during right hand double clicking.** The solid line at the top shows the signal from the trigger incorporated in the left mouse key, giving the time of activation of the key.

Four subjects showed continuous activity with only a weak or no association between the small fluctuation in IFR and the timing of DC For an example see **Figure 3**, showing the IFR for the two continuously active MU identified in ID 9. In general MFR for these MUs was low, and no pronounced changes in firing rate were present during the DC. For each subject a general common trend in IFR modulation was seen for all the identified MUs resulting in CCCs among MU averaged IFR for these subjects ranging from 0.40 to 0.81.

**FIGURE 4:**
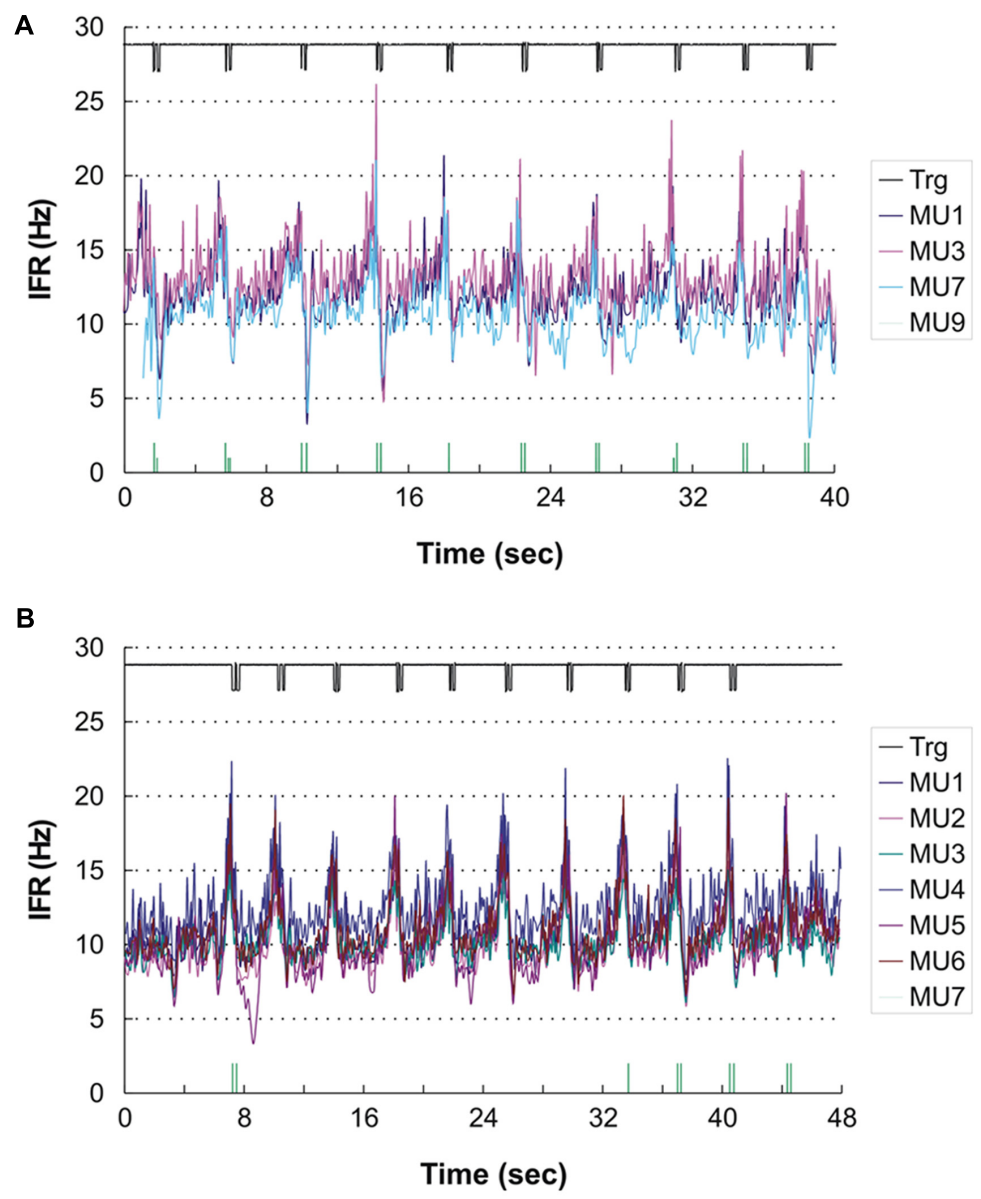
**(A,B)** Instantaneous firing rate plot showing the firing pattern of the identified MUs in subject 12 during right hand **(A)** and left hand **(B)** double click (DC). As MUs cannot be traced across recordings the MU numbering is arbitrary. The solid line at the top shows the signal from the trigger incorporated in the mouse key, giving the time of activation of the key. The vertical bars in the bottom of **(A)** and **(B)** present the inter firing bar plot for MU 9 and MU 7, respectively, as their intermittent firing pattern is not suited for an IFR presentation. MU doublets are illustrated by high bars.

For the remaining four subjects a common pattern of IFR with large modulation in firing rate with a clear temporal relation to the DC was present. The lowest level of IFR was found right after the DC with a gradual increase in firing rate for all identified MUs until the next DC. As an example, see **Figure 4A** showing the IFR for the active MUs identified in subject 12 and the temporal relation to the DC shown in the top. The CCC among MU averaged IFR for the subjects showing IFR modulation ranged from 0.64 to 0.96.

In three subjects double discharges were identified. In one of the subjects the modulation in IFR was accentuated by the recruitment of a MU almost only being active with doublets (shown in the bottom of **Figure 4A**). All 16 doublets were coincidental with the peaks in IFR of the concurrently active MU. Another two subjects showed a few occasional doublets, which however, did not seem to be timely related to the DC.

**FIGURE 5:**
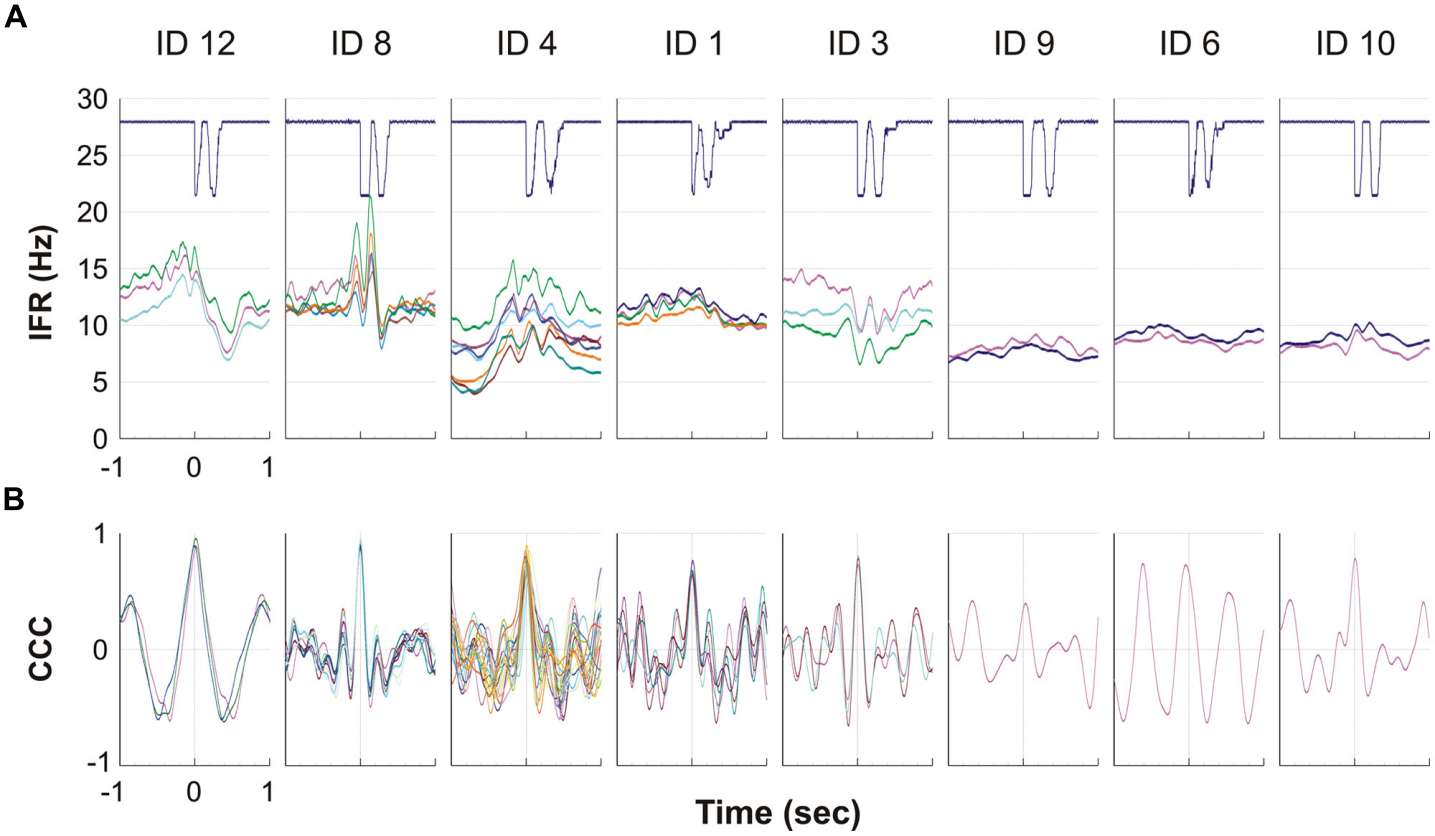
**(A,B)** The eight graphs, one for each of the eight subjects with MUs with continuous activity, show the averaged firing rate for each MU during the ipsilateral DC. The solid line at the top shows the signal from the trigger, giving the time of activation of the mouse key, which is used to align DC before averaging the firing rate. Each of the lines below represents a MU and shows the IFR averaged for the 10 DC. **(B)** For each of the eight subjects the cross correlation function is given for the averaged IFR of pairs of all MU presented in **(A)**. Please note that the time axis is here giving the 1 s time shift back and forth for the cross correlation function.

For all eight subjects showing MU activity during DC, the result of the spike triggered averaging of the IFR for all the identified continuously active MUs during the 10 right hand DCs is presented in **Figure 5A**. For four subjects (left side of **Figure 5A**) a more or less pronounced anticipatory increase in IFR can be seen, Subjects 3 showed a special DC related modulation with rather a decrease in IFR, while the remaining three subjects (right side in **Figure 5A**) showed a very modest or no anticipatory increase in IFR in relation to the timing of double clicking. Further, the lack of modulation in IFR for these three subjects also was associated with a low firing rate staying below 10 pps. In **Figure 5B** the cross correlation function of the averaged IFR for all MU pairs in each recording is shown. The shared common synaptic input is clearly visible as a common modulation of the averaged IFR of all identified MUs from each recording site as well as evidenced by the high peak CCC shown below. Mean time shift for peak CCC among subjects was 3 ms and ranged from -39 to 30 ms (See **Table 2**; **Figure 5B**).

### CONTRALATERAL DC

For the left index finger DC task contralateral activity was found in the right trapezius muscle for 4 of the 12 subjects but only two subjects showed continuously active MUAPTs. In total 15 MUs were identified and 10 of these showed continuous activity with MFR from 10.1 to 16.1 pps. The number of identified firings in the MUAPT ranged from 311 to 603 (see **Table 1**). Fourteen MUs were continuously active in the 2 s period surrounding the DC and were included in the averaged IFR used for the calculation of CCC presented in **Table 2**. Peak CCC was as a mean 0.90 and ranged from 0.82 to 0.99 with a mean time lag of 5 ms ranging from 18 to 39 ms for the two subjects.

A temporal relation of the IFR and DC was seen for two of the subjects, while the two other showed a weak or no association. Subject 12 who showed peak related doublets for ipsilateral DC also showed nine peak related doublets during the contralateral DC (see **Figure 4B**). In this recording the anticipatory activity can clearly be seen in the end of the recording, where the subject obviously is preparing for the DC number 11 before being stopped by the experimenter.

**FIGURE 6:**
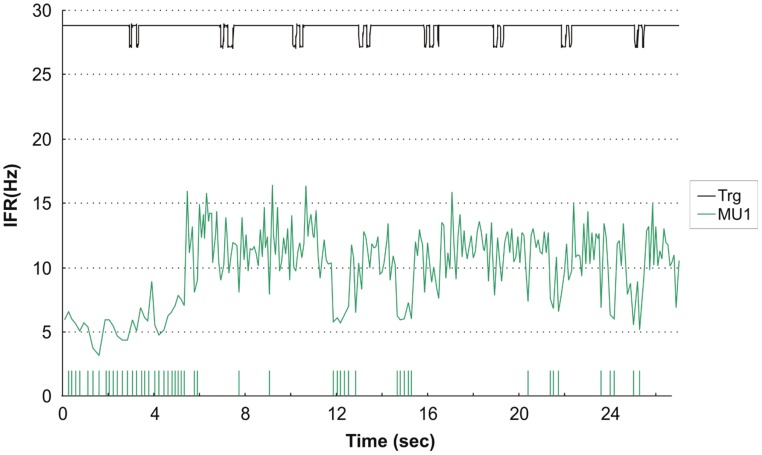
**Instantaneous firing rate plot showing the firing pattern of one identified MU in subject 2 during left hand double clicking.** The solid line at the top shows the signal from the trigger incorporated in the mouse key, giving the time of activation of the key. After 29 doublets the MU transitioned from repetitive doublet firing to single spike firing pattern. IFR is for the whole MU action potential trains (MUAPT) calculated disregarding the inter-doublet spike interval. The vertical high bars in the bottom indicate the occurrence of doublets. Note, that the doublet firing rate is markly lower than the single spike firing rate and this is evident at all remissions to doublet firings in the recording.

Interestingly, one of the subjects who presented with complete silence during right index finger movements had one active MU during left index finger DC with a rather distinct firing pattern. The MU activity pattern is presented in **Figure 6** and shows initial repetitive doublet firings according to the criteria of Bawa and Calancie (1983). The doublets showed no relation to the timing of the finger movement, and after 29 doublets firing the MU transitioned from repetitive doublet firing to single spike firing pattern with an abrupt rise in firing rate compared to the firing rate of the doublets disregarding the inter-doublet spike interval. During the remaining recording the MU occasionally reverted to short doublet series, interrupting the single-spike firing.

## DISCUSSION

While there was no difference in the overall activity recorded as surface EMG, three different responses on a single MU level were seen in the trapezius muscle during DC: no activity, activity weakly related and activity strongly related to the mechanical task of fast finger movements. Interestingly, in each recording all the averaged IFR of the identified MUs followed the same pattern with pairs of MUs showing high peak CCC indicating a shared synaptic common drive.

A clear connection between the mechanical performance of the index finger and the MU firing rate modulation in the ipsilateral trapezius was seen in four of the subjects. This supports the idea of the mechanically related multi-joint motor program activating the trapezius muscle anticipatory to the planned movement of the index finger. However, for two of the subjects the same pattern was seen for contralateral index finger movements with no obvious biomechanical reason as has also been reported for the extensor digitorum communis muscle during finger movements (Søgaard et al., 2001). This may be a pre-programmed bilateral overflow phenomenon of an anticipatory motor program aiming at the provision of a stable shoulder girdle in order to perform precise finger manipulations. In the present study we do not have simultaneous intramuscular recordings from both shoulders to reveal whether an increase in activity before each DC is equally pronounced in both shoulders. If this is the case, it may be evoked by a generally increased alertness before each DC performance. Further, in the present study no simultaneous recordings were performed to reveal the temporal relation in activation for the agonistic extensor digitorum communis actually lifting the index finger and trapezius to indicate a multi joint activation program.

A MU firing pattern only weakly related to the actual mechanical task to be performed was seen in four subjects, which may be interpreted as attention related background activity independent of the finger activity. Interestingly, four subjects apparently were able to perform right hand DC without any identified MU activity although low threshold MU were active during the standard contractions before and after the DC task.

Since the highly selective electrode only records activity within a small muscle volume in the vicinity of the recording area, the wire electrode may just happen to be in a silent subpart of the muscle for this task. A visual inspection as well as the CCC shows a clear dependence between activity patterns in MUs from the same recording site. As each recording site is in a specific subject, results are here given on a subject level, keeping in mind that differences may just represent the difference in activity patterns between trapezius muscle subparts. Indeed, earlier studies on single MU activity in the trapezius muscle have indicated that such a spatial inhomogeneity is likely to exist with a task specific selective activation (Stephenson and Maluf, 2010). Of note is that all subjects had similar level of surface EMG representing the summarized activity of active MUs within the muscle volume below the recording surface electrodes. This strongly indicates that each intramuscular recording may rather be considered as an activity pattern in a specific recording area of the muscle than as a difference between subjects.

Both the attention related MU activity in the trapezius muscle with constant low MFR as well as the activity in MUs with large modulation of IFR in clear relation to the mechanical performance of the index finger could be avoided from a biomechanical point of view as the provided support of the elbow should minimize the need for shoulder joint stabilization. While the MUs with no modulation in firing rate generally are activated at a low firing rate level, the modulating MUs generally are activated at higher MFR with peak levels. Both activity patterns in the perspective of prevention of myalgia represent activation patterns that are not caused by a biomechanical demand but evoked or maintained just by the planning and performance of the movement of the index finger. Such activity may metabolically compromise intracellular homeostasis and eventually impair Ca^2+^ transients resulting in elevated cytosolic [Ca^2+^] (Westerblad et al., 1991; Gissel, 2000). This process may promote breakdown of the muscle membrane and a leaky cell membrane may cause muscle metabolites to stimulate the nociceptive free nerve endings in the interstitium (for more details see Sjøgaard and Søgaard, 1998).

The present study was not designed to specifically evoke doublets, but doublets were identified for 4 subjects out of 12. One subject showed doublets in the right trapezius muscle related to the fast movements of both the ipsi- and contralateral index finger, in two other subjects MUs occasionally showed doublets unrelated to DC and one subject during contralateral DC showed repetitive doublets. This indicates that doublets are not only linked to demand of high contraction velocity to lift the finger as earlier reported for the finger extensors (Sjøgaard et al., 2001; Søgaard et al., 2001) but also can be evoked by the need of fast stabilization of the shoulder joints and that this may be an integrated part of normal motor control for some subparts of the motor neuron pool.

Stimulation studies have shown, that a doublet can potentiate the twitch force and in good agreement, this activation strategy in voluntary contractions is mainly found when high rate of force development and/or shortening velocity are required (Kossev and Christova, 1998; Thomas et al., 1999; Cheng et al., 2013). In addition to the large initial rate of force development, also a much larger force is maintained during the following excitations, a phenomenon termed catch like properties (Sandercock and Heckman, 1997). This implies that a doublet may impose a higher tension during the subsequent activity than indicated by the actual firing rate of the MU.

Since the earliest description of doublets by Denslow (1948) the origin has been debated and so has the distinction between the occasionally evoked doublets and the repetitive doublets (Bawa et al., 2000; Kudina and Andreeva, 2010). However, as recently stated doublets are rarely recorded during voluntary muscle activity in human subjects (Piotrkiewicz and Kuraszkiewicz, 2014). It is therefore interesting that in the present study simulating free living conditions with no feedback to the subject, a MU presented a firing pattern with repetitive doublets transitioning from stable doublets to stable single spikes firing. Such a firing behavior was earlier described by Bawa and Calancie (1983) and later provoked with MU feedback by Kudina and Andreeva (2010). Repetitive doublets followed by a jump in firing rate could be considered as the bistable firing behavior characterizing a plateau potential. Human plateau potentials have been suggested as an underlying cause of the sustained low level activation of some MU (Kiehn and Eken, 1997; Gorassini et al., 1998). Interestingly, two other studies have reported similar observations of plateau potential like behavior in trapezius MU, one of them actually during almost similar conditions of finger movements (Westad et al., 2004; Stephenson and Maluf, 2010).

One of the strengths of the present study is the use of an averaged IFR profile summarizing detailed information on MUAPTs from 10 repetitions of the studied finger task in the characterization. To our knowledge, this approach to a robust description of the IFR modulation during a certain task has not earlier been conducted in other studies. Further, the task is a functional unrestricted task performed with no feedback on the discharge rate provided for the subject. Therefore, the recorded MU activity for each subject may to a high degree reflect the motor activation pattern used in daily life. A limitation is the use of highly selective wire electrodes providing information on activity in only a small localized part of the upper trapezius muscle that may not be representative for the whole subpart of the Trapezius muscle in each of the subjects. Further, it should be acknowledged that the study is based on a limited number of MUs only allowing an exploratory approach.

## CONCLUSION

Several rather distinct MU IFR patterns were identified in the right trapezius muscle during DC with ipsi- and contralateral index finger, in spite of similar low level of surface EMG activity in all subjects. In 75% of the subjects, an attention related IFR activity pattern and/or index finger movement related activity pattern for ipsi- or contralateral DC were seen. In 25% of the subjects doublets were seen, for one subject temporarily related to peak activity in concurrently active MU during both ipsi- and contralateral DC and for the other two subjects as occasional events. The continuous activity of some MUs, the contralateral central drive overflow, and the doublet phenomenon possibly causing an increase in mechanical MU loading may contribute to an explanation of computer work related development of fatigue and myalgia in the trapezius muscle.

## AUTHOR CONTRIBUTIONS

All authors substantially contributed to the conception and design of the work and in the interpretation of data. All authors participated in the data acquisition led by Anne K. Blangsted as well as in the analysis led by Henrik B. Olsen. Karen Søgaard and Henrik B. Olsen drafted the work and all authors revised it critically for important intellectual content. All authors finally approved the version to be published.

## Conflict of Interest Statement

The authors declare that the research was conducted in the absence of any commercial or financial relationships that could be construed as a potential conflict of interest.
